# Telemedicine for COVID-19 management in Brazil: outcomes and health system implications from a prospective cohort study

**DOI:** 10.1590/1678-9199-JVATITD-2025-0030

**Published:** 2025-12-15

**Authors:** Ana Silvia Sartori Barraviera Seabra Ferreira, Cassiana Mendes Bertoncelo Fontes, Lehana Thabane, Carolina Russo Simon, João Pedro Pereira Caetano de Lima, Jean Carlos Possidônio da Silva, Benedito Barraviera, Raul Borges Guimarães, Pasqual Barretti, Rui Seabra Ferreira

**Affiliations:** 1 Center for the Study of Venoms and Venomous Animals (Cevap), São Paulo State University (Unesp), Botucatu, SP, Brazil.; 2 Department of Health Research Methods, Evidence, and Impact, Faculty of Health Sciences, McMaster University, Hamilton, Ontario, Canada.; 3 Research Institute of St Joe’s Hamilton, St Joseph’s Healthcare Hamilton, Hamilton, Ontario, Canada.; 4 Department of Geography, School of Sciences and Technology, São Paulo State University (Unesp), Presidente Prudente, SP, Brazil.; 5 Graduate Program in Geography, Faculty of Science and Technology, São Paulo State University (Unesp), Presidente Prudente, SP, Brazil.; 6 Botucatu Medical School (FMB), São Paulo State University (Unesp), Botucatu, SP, Brazil.

**Keywords:** Telehealth, Teleconsultation, Remote consultation, Public health, COVID-19

## Abstract

**Background:**

The COVID-19 pandemic exposed vulnerabilities in traditional disease surveillance systems, particularly in data reporting and contact tracing. Telemedicine emerged as a promising approach to expand remote access to healthcare. This study aimed to evaluate a newly implemented telemedicine system designed to manage patients with COVID-19, reduce hospital overload, enable early case detection and isolation, ensure rapid response to clinical deterioration, simplify medical records, and provide ongoing patient support.

**Methods:**

A prospective cohort study was conducted using the E-care telemedicine system to assist adult patients presenting with COVID-19 symptoms at a Brazilian university between June 2021 and June 2024.

**Results:**

The E-care system delivered care to 6,129 patients, predominantly female, white university students. Physicians attended over 80% (4,903/6,129) of patients and prescribed medications to nearly 28% (1,411/5,041). Medical certificates for time off work were issued to 43% (2,635/6,129) of participants. COVID-19 tests were recommended for approximately 24% of patients, with a positivity rate above 81% among those who returned results. Only 66 patients (1.2%) required in-person care, and no COVID-19-related deaths were reported. Patient satisfaction was high, with 96% (5,584/6,129) expressing satisfaction or high satisfaction with the service.

**Conclusions:**

This study provides robust evidence supporting the successful implementation of a telemedicine system for managing COVID-19 cases. The large number of users highlights an unmet demand for virtual healthcare. Telemedicine was rapidly adopted, achieved high patient satisfaction, and contributed to reducing hospital burden, promoting early detection, and minimizing in-person consultations. These findings reinforce the value of telemedicine as an essential tool for health systems and policymakers to strengthen care delivery beyond the pandemic.

## Visual Abstract

**Figure:**
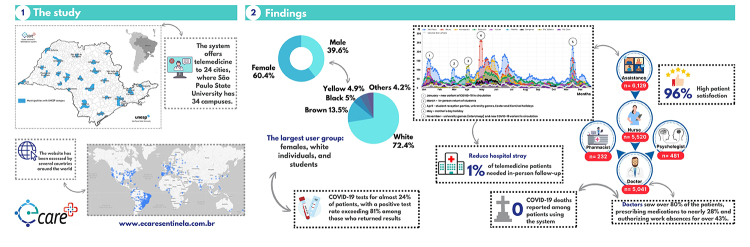
Graphical abstract

## Background

The sudden emergence and global spread of the Sars-Cov-2 virus in late 2019/early 2020 (COVID-19) severely strained global health systems. The pandemic caused significant disruptions in essential healthcare delivery, leading to hospital overcrowding. In response, governments worldwide, including Brazil, acted swiftly to promote and simplify the use of telemedicine. This aimed to facilitate patient access to medical consultations, prescriptions, and reports without the need for in-person visits.

The rapidly evolving world of internet-enabled healthcare necessitates robust investment and rigorous research of telemedicine systems. This proactive approach is crucial to navigate advancements, understand their impact on healthcare systems public and private, and ultimately optimize the delivery of high-quality care for everyone [1].

Telemedicine, by eliminating travel requirements, exemplifies this optimization offering agility, increased service capacity, and reduced risk of exposure to diseases for both patients and healthcare professionals. Patients benefit from avoiding unnecessary hospital visits, minimizing the spread of infectious diseases [2], especially during outbreaks [3]. Like many countries, Brazil lacked up-to-date legislation to formally integrate telemedicine into the healthcare system. 

Brazil has one of the world’s largest and most complex public health systems - the SUS (Brazil’s Unified Health System) - which provides comprehensive medical care, ranging from basic checkups to complex organ transplants, for more than 215 million citizens. However, the COVID-19 pandemic severely tested its resilience [4]. In response, Brazil implemented several measures to curb viral transmission, including quarantines, restrictions on public gatherings, and the closure of schools, churches, workplaces, and most businesses. The surge in cases also required a rapid and substantial expansion of hospital capacity, particularly in intensive care units (ICUs) [5].

The early stages of the pandemic raised concerns about Brazil's capacity to handle the surge in demand for hospital and ICU beds. As Bigoni *et al.* [6] point out, ICU occupancy peaked in the second half of 2020. This overwhelming need for critical care unfortunately led to a decline in non-COVID-19 healthcare procedures. It is important to note that this was not unique to Brazil. Countries like Italy, Spain, and several regions in Asia faced similar challenges and reported a decline in elective procedures during the pandemic [6].

Consequently, the COVID-19 pandemic exposed vulnerabilities of the traditional disease monitoring systems, particularly data reporting and contact tracing. These systems often buckled under the strain of exponentially rising cases. Telemedicine emerged as a powerful tool in this context, enabling remote access to healthcare, especially during public health emergencies like COVID-19 [7]. Since teleconsultations were legally prohibited in Brazil until 2020, there are no existing studies that evaluate their impact on patients.

Walley *et al.* [8] found that both patients and general practitioners reported high satisfaction with telemedicine during the COVID-19 pandemic. Patients appreciated the ability to receive care while minimizing their risk of infection, aligning with public health guidelines. Practitioners valued the reduced risk of contracting COVID-19, enabling them to continue serving their patients' needs. Younger patients, accustomed to technology, were particularly well-suited to telemedicine [8].

This study presents a successful, fully online multidisciplinary care system implemented in Brazil. Staffed by a dedicated team of healthcare professionals, the system provided real-time virtual consultations for patients with suspected Sars-Cov-2 infection. Its main objectives were to:

- Reduce hospital burden: by offering remote medical care and patient monitoring.
- Enable early identification and isolation: teleconsultations supported presumptive diagnoses, allowing for timely intervention and isolation to prevent further transmission.
- Ensure a swift response to clinical deterioration: when a patient’s condition worsened, the team could promptly arrange in-person evaluations, hospital admissions, or emergency referrals.
- Streamline documentation: the service simplified the process for obtaining medical prescriptions and sick-leave certificates.
- Provide ongoing support: the team offered continuous guidance and follow-up to patients and their families throughout the illness.

## Methods

### Context

**Figure 1.**
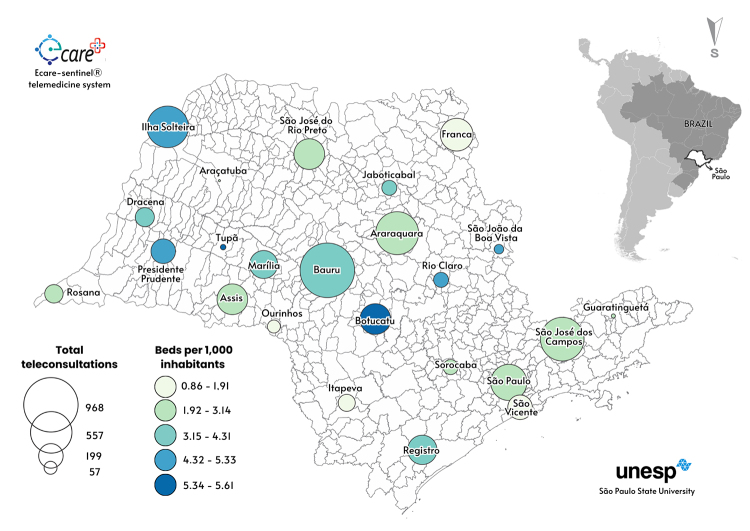
Geographical distribution of São Paulo State University campuses, Brazil, and number of beds per 1,000 inhabitants.

This prospective cohort study evaluated the use of the E-care telemedicine system between June 2021 and June 2024. As inclusion criteria it followed 6,129 adult patients with COVID-19-like symptoms, including workers, students, and their families, all affiliated with São Paulo State University (Unesp) spread in 34 campuses across 24 cities of São Paulo State, Brazil (Figure 1). The reporting adhered to the Revised Standards for Quality Improvement Reporting Excellence (SQUIRE 2.0) [9, 10].

### Setting

Amid the disruptions caused by the COVID-19 closures between 2020 and 2022, São Paulo State University (Unesp) - a public Brazilian institution with more than 51,800 students and 11,400 employees, including faculty and administrative staff - implemented a telemedicine system to maintain access to healthcare services. This innovative initiative provided the university community with multidisciplinary care from doctors, nurses, pharmacists, and psychologists through virtual consultations. By enabling remote appointments, the system helped reduce unnecessary in-person visits to outpatient clinics and hospitals.

### Intervention

The E-care telemedicine system provided COVID-19 care for a university community and an advertising campaign raised, encouraging those with symptoms or potential exposure to use it. To ensure optimal care, the system utilized a staged approach for patient consultations. An initial screening and multidisciplinary support were conducted by a nurse to determine if a doctor's evaluation was needed. Psychologists and pharmacists were also available for a holistic approach. The E-care system's patient portal functioned as a central information hub, offering users targeted educational materials based on scientific literature and team contact information.

Patients filled out a symptom survey based on a COVID-19 decision tree to streamline assessment by nurses and doctors (Additional file 1). This approach allowed healthcare providers to quickly assess a patient's condition and determine the appropriate course of action. Before entering the virtual waiting room, patients reviewed and signed a digital consent form.

Finally, patients waited in an online space with informative COVID-19 videos while a virtual receptionist directed them to their consultation. All user information was encrypted and securely stored. Consultations (except psychology) were recorded for continuity of care.

### Measures and analysis

System access and analyzed data

Between June 2021 and June 2024, the E-care telemedicine system recorded 6,129 unique visitors, each of whom received consultation and guidance from the multidisciplinary care team. The consultations were available only from 08:00 to 18:00. Therefore, website traffic outside these hours was excluded from the study.

The information collected from website users included demographics, medical history, and consultation details to better understand their health needs.

Teleconsultation analysis

To assess the effectiveness of the telemedicine consultations, these consultations were analyzed by researchers’ various aspects of the care provided by the multidisciplinary team as detailed breakdown:

- Nurse activity: number of patients seen by nurses and the number of patients they referred to doctors for further evaluation.
- Team collaboration: frequency of inter consultations between doctors, psychologists, and pharmacists to ensure comprehensive care for patients.
- Treatment provided: number of prescriptions issued by doctors, work absence certificates provided, and COVID-19 test requests submitted.
- Patient outcomes: results of COVID-19 tests (positive/negative) and the number of patients who required in-person care after being seen by the multidisciplinary team.

Data analysis and visualization

The teleconsultation data was analyzed to understand the geographical patterns of usage. Initially, the data was cleaned and organized using a spreadsheet program such as Microsoft Excel 16.7. After this initial stage, the information about cities and zip codes was transformed into geographical coordinates (latitude and longitude) for each consultation. The geocoded information was then used as the basis for geographical analysis. Then, specialized GIS (Geographic Information System) software (ArcGIS Pro 3.2.2) was used, along with PhilCarto 5.01 mapping software, to create maps showing the trends and geospatial patterns of the teleconsultations.

Enhancing the analysis with geography and thematic maps

Thematic maps were developed to visualize the impact of the project in different municipalities where the university is based. These maps show how the project has affected Brazil's public health system, because by making teleconsultations a “localized phenomena” it is possible to understand how many people have not had to attend in-person care in this regard, previous studies have shown that maps are powerful tools for analyzing these spatial trends and assisting in health analyses [11]. Critical cartographical visualization, together with advances in geographical analysis, have enabled maps to become both informative and analytical documents. Through a set of cartographic techniques, results can be observed that effectively communicate the geographical scope and influence of the project [12].

### Ethical considerations

To ensure compliance with ethical standards, the project was submitted to, reviewed, and approved by the Research Ethics Committee of the Botucatu Medical School (FMB), São Paulo State University (Unesp), on May 1, 2020 (approval number: 4,002,318). Subsequently, an amendment to the project was submitted and approved on April 19, 2021 (approval number: 4,659,395).

## Results

From June 2021 to June 2024, the E-care telemedicine system provided services to 6,129 patients. Notably, receptionists within the virtual waiting room were able to efficiently resolve inquiries for 609 patients without the need to continue receiving care from the medical team.

To ensure optimal care, the entire multidisciplinary team received comprehensive training on COVID-19 management. Additionally, on-duty team members remained connected through a dedicated chat channel within the administrative area. This channel facilitated real-time communication, allowing for the sharing of support links, and resolving common questions directly (9.93% of consultations, n = 6,129). Frequently addressed topics in the chat included work leave guidelines, safe workplace return protocols, mask and hand hygiene practices, isolation procedures, free COVID-19 testing locations, and operational hours. By addressing these inquiries promptly, patients avoided unnecessary visits to health units or emergency rooms, thereby minimizing congestion and potential exposure risks.

**Figure 2.**
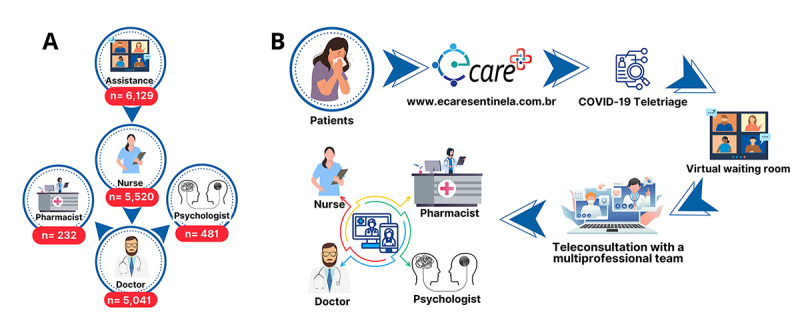
(A) Patient flowchart for E-care teleconsultations (June 2021 to June 2024) showing the complete journey of patients seeking multiprofessional teleconsultations for COVID-19. (B) E-care workflow.

Figure 2 A  and 2B highlights the important role specialist nurses played in initial patient assessment and screening and E-care Workflow. They addressed a wide range of needs, including medical, psychological, and pharmaceutical. The nursing team's expertise is further emphasized by their successful management of 479 patients (8.68%, n = 5,520) without requiring doctor referrals.

In most cases, physicians served as the primary caregivers (n = 5,041, 85.86%), with only a minimal need for consultations with pharmacists (n = 232, 4.6%) and psychologists (n = 481, 9.54%). Notably, all patients who received care from doctors had been previously seen by nurses. Likewise, all patients who consulted with pharmacists or psychologists had prior consultations with both doctors and nurses. Interestingly, there were no instances where a patient referred to a psychologist had also been referred to a pharmacist, and vice versa.

Table 1 shows a comprehensive analysis of 6,129 patients treated by E-care encompassing their baseline characteristics and common occurrences. Female patients, white individuals, and students made up the largest portion of those treated. Hypertension (7.6%) and diabetes (2.6%) emerged as the most prevalent chronic conditions. Runny nose/nasal congestion (72.5%) and dry cough/itchy throat (72.9%) were the most frequently reported clinical symptoms, while respiratory difficulty (12.6%) was the least common. Interestingly, the prevalence of respiratory difficulty closely mirrored the percentage of patients who identified as smokers (11.8%), suggesting a potential link between these factors. Self-medication emerged as a sentinel event, practiced by 3% of the patients.

**Table 1.** Demographic baseline characteristics and common occurrences among 6,129 patients assisted by E-care telemedicine system between June 2021 and June 2024.

Variables		Total	
		n	%
**Gender**	Male	2,426	39.58
	Female	3,703	60.42
**Race/ethnicity**	White	4,439	72.4
	Brown	826	13.5
	Black	305	5.0
	Yellow	299	4.9
	Indigenous	7	0.1
	Others	29	0.5
	NI	224	3.7
**Institutional bond**	Student	2,445	39.9
	Professor	452	7.4
	Administrative technician	1,384	22.6
	Outsourced	98	1.6
	NI	1,750	28.6
**Chronic diseases**	Hypertension	369	6.0
	Diabetes	158	2.6
	Respiratory diseases	467	7.6
	Neurological diseases	53	0.9
	Cardiovascular diseases	54	0.9
	Liver diseases	17	0.3
	Kidney diseases	36	0.6
	Obesity	48	0.8
	Smoking-related diseases	40	0.7
	HIV	3	0.05
	Cancer	8	0.1
	Bone marrow transplant history	6	0.1
**Habit/symptoms**	Smoker	725	11.8
	Fever	1,862	30.4
	Runny nose or nasal congestion	4,611	75.2
	Dry cough or itchy throat	4,466	72.9
	Headache	4,067	66.4
	Sore throat	4,010	65.4
	General malaise, fatigue, or muscle pain	4,078	66.5
	Difficulty breathing	773	12.6
**Sentinel events**	Self-medication	183	3.0
	Inadequate medication storage	85	1.4
	Inadequate medication disposal	88	1.4
	Use of inappropriate medication	19	0.3
	Non-adherence to treatment	31	0.5
	Adverse reaction	17	0.3
	No validity check	13	0.3
	Consumption of medicines after their expiration date	29	0.5
	The patients were not isolated until the test results were reported	1	0.02
	Neglect of protective measures against COVID-19	1	0.02
	Symptomatic and/or non-isolated positive	14	0.2

NI: not informed; HIV: human immunodeficiency virus

Among 5,041 patients seen by doctors, 1,414 (28.05%) received prescriptions. Doctors also authorized absence from work for 2,186 patients (43.36%) and recommended COVID-19 exams for 1,208 patients (23.96%). Of those who received exam recommendations (1,208), just 752 patients returned their results, with 613 (81.5%) testing positive and 139 (18.5%) testing negative (Table 2).

**Table 2.** Number of patients undergoing multiprofessional care, different recommendations, and consequences.

Patient variables	n	(%)
Total number of patients seen by doctors	5,041	100.00
Patients who received doctor's prescriptions	1,414	28.05
Patients who received a certificate of absence from work	2,186	43.36
Patients who received COVID-19 exam's solicitation	1,208	23.96
Patients who returned COVID-19 test results	752	100.00
Tested positive for COVID-19	613	81.50
Tested negative for COVID-19	139	18.50
Total of patients treated by the multiprofessional team	5,520	100.00
Patients sent for in-person care	66	1.20

Only 66 out of the 5,520 patients treated by the healthcare team were referred by the multidisciplinary group for an in-person physical assessment. These referrals were prompted by the identification of:

- Symptoms warranting in-person assessment: shortness of breath, wheezing or chest pain, chest pressure, fatigue, dyspnea (difficulty breathing), respiratory discomfort, myalgia (muscle aches), or persistent COVID-19 symptoms.
- Clinical reasons for in-person evaluation: to distinguish between bacterial and viral infections; to examine the oropharynx (throat); to assess lung and kidney function; to rule out secondary infections; to investigate significant weight loss during COVID-19; to determine the cause of isolated fever; to assess changes in breathing pattern; to check for dehydration; to diagnose pneumonia or bacterial tonsillitis; to rule out a hypertensive crisis (a sudden, severe increase in blood pressure); or to diagnose dengue fever or sinusitis

Encouragingly, no COVID-19-related deaths were reported among patients treated by the system during the study period.

The website access data analysis revealed potential correlations between regional case surges and specific events (Figure 2). Spikes in website traffic and teleconsultations coincided with national holidays and university gatherings. For instance, January 2022 witnessed a peak likely associated with the circulation of the Omicron variant. 

Similarly, peak 2 aligned with the return of Unesp students to in-person classes in March. April saw a surge coinciding with student welcome parties, university games, and the Easter holiday, while peak 3 likely reflected the impact of Brazil's Carnival celebrations (initially modified due to February's case increase). May's peak 4 potentially stemmed from Mother's Day gatherings, a time when many students travel home. Finally, peak 5 in November aligned with both the "InterUnesp" university games and the emergence of a new Omicron variant in Brazil. 

**Figure 3.**
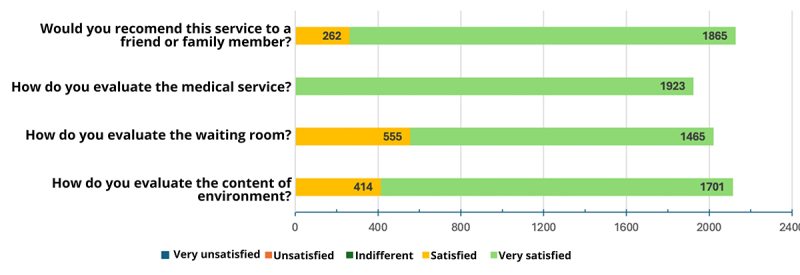
E-care website access peaks correlated with events in 2022.

Figure 3 depicts monthly website access trends for the E-care telemedicine system in 2022. The highlighted peaks coincide with significant events such as national holidays, university gatherings, and the circulation of new COVID-19 variants. 

**Figure 4.**
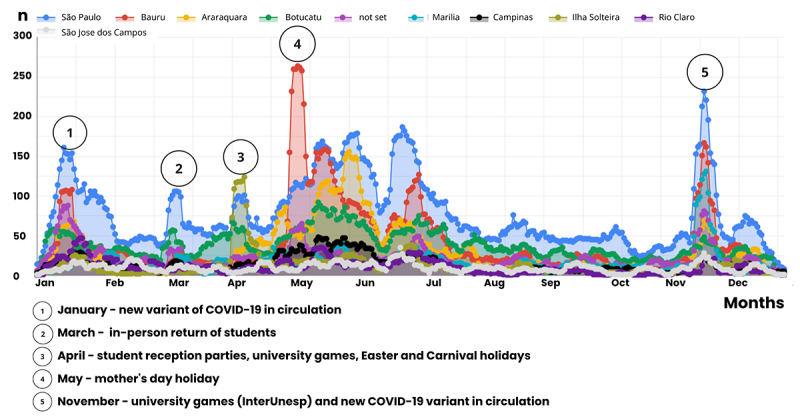
E-care telemedicine system patient satisfaction survey results showing the breakdown of patient satisfaction responses collected through a post-teleconsultation survey.

Following each teleconsultation, patients received a satisfaction survey via email. The results were overwhelmingly positive, with 96,4% (n = 2188) of respondents indicating satisfaction or high satisfaction with the E-care telemedicine system (Figure 4).

Patients also provided valuable feedback through comments and suggestions in response to the last research question.

## Discussion

The COVID-19 pandemic accelerated the global adoption of telemedicine, with general practitioners and patients rapidly transitioning from in-person consultations to digital remote care. Evaluating the impact of this shift on patient care, healthcare providers, patient and carer experiences, and health systems is crucial [13]. The E-care telemedicine system evaluation highlighted the importance of multidisciplinary care for COVID-19 patients, potentially to help prevent future comorbidities.

The World Health Organization saw telemedicine as a tool to improve healthcare access, reduce costs, and expand services - especially for remote and underserved communities. The COVID19 pandemic, however, amplified this vision highlighting the urgent need for innovative solutions to alleviate pressure on global healthcare systems, manage rising demand for services, and minimize the risk of transmission of infectious diseases in outpatient clinics. 

Telemedicine emerged as a crucial tool to address these challenges [14]. Teleconsultation platforms, an environment where patients can schedule remote consultations with doctors with whom they have had no prior relationship, or even receive immediate care, represented a significant change in the medical consultation market. This fact has generated a different relationship between doctors and patients who often use these platforms only for advice [1].

The digital platforms prepared for medical care have been available around the world for a long time, although few countries had the courage to regulate them. In Brazil, it was no different, because since 2002 the country has tried to implement telemedicine and just in 2022 was defined and regulated medical services mediated by technology. 

Phuong J *et al*. [15] proposed the following recommendations to a telemedicine system: design technology and systems using accessibility and value sensitive design principles; support a range of technologies and settings; support multiple and diverse users; and support clear paths for repair when technical systems fail to meet users’ needs [15]. The COVID-19 pandemic impact has revealed the need for a rapid and comprehensive approach to patient care, a multiprofessional service as offered by E-care telemedicine system could, if used worldwide, have prevented not only the immediate effects of the virus, but also the potential for future complications that these patients could face during and after the pandemic.

The predominance of students using digital healthcare may be due to their greater comfort with technology or their lack of access to a private healthcare system. Baseline characteristics and common occurrences among the 6,129 patients treated by E-care telemedicine system showed valuable information about the main signs and symptoms that led individuals to seek medical services during the COVID-19 pandemic. Furthermore, it was possible to diagnose silent and non-symptomatic diseases such as hypertension and diabetes mellitus. It was also possible to estimate a diagnosis of frequent habits in our students, such as tobacco use. The main sentinel event observed was self-medication, a widespread culture in that population.

Also, it was observed that only 66 (1.2%) patients required referral for in-person medical care. This finding helps validate the efficiency of telemedicine systems. By successfully addressing the needs of over 6,000 patients remotely, E-care telemedicine system has helped to reduce the burden on healthcare systems by minimizing in-person visits and minimizing the spread of COVID-19 among patients and healthcare workers.

Telemedicine is not a one-size-fits-all solution. While telemedicine offers benefits, its adoption is challenged by a community's health status and, primarily, its cultural ability to use digital equipment. This study's sample population may be a limitation. A Chinese study found that telemedicine, combined with AI and mHealth, is a cost-effective way to prevent blindness in older adults [16].

E-care telemedicine system data identified interesting correlations between website activity and real-world occurrences. Site traffic spikes coincided with significant events such as national holidays, university meetings, and outbreaks caused by new variants of COVID-19. It should be noted that Mother's Day, university games, and the occurrence of new variants of the virus had a direct impact on the increase in transmissibility. This new information was valuable and provided university management with insights to guide academic and public health decision-making and, if necessary, the allocation of financial resources.

The multidisciplinary team responsible for the E-care telemedicine system achieved a 96% user acceptance rate, a finding that significantly supported its initial and subsequent full-scale implementation. This team's enthusiasm was motivated by the responses obtained in the questionnaires submitted to users because all participants reported being satisfied or very satisfied with the service corroborated by a systematic review conducted in 2023 by Alashek and Ali [17]. The authors have analyzed 24 studies and found high satisfaction with telemedicine services among users in the United Kingdom during the COVID-19 pandemic [17].

Several studies have demonstrated high satisfaction with telemedicine services. Notably, in studies focusing on pediatric patients, where information was provided by parents and caregivers, satisfaction levels were exceptionally high. Darr *et al.* [18] obtained rates above 98% and Makhecha *et al*. had 96% satisfaction [18, 19]. Similarly, Kaur *et al.* [20] reported a 97% satisfaction rate with the quality of the service among patients in general. Others authors have shown the advantages of telemedicine in various specialties as a differentiating and important factor for patient satisfaction [21-23].

Gomes Rodrigues *et al*. [24], have demonstrated the potential of telemedicine to significantly improve patient outcomes, reduce healthcare costs, and alleviate the global diabetes burden. Their findings underscore the importance of integrating telemedicine into public health policies, especially in developing nations like Brazil.

Telemedicine has significant potential but faces barriers like accessibility, cultural beliefs, and financial cost. While it has shown benefits during the pandemic, challenges like technological limitations and the need for qualified professionals persist. Positive experiences can increase acceptance of telemedicine, but its widespread adoption may require addressing these barriers [25, 26].

Overcoming these challenges, for the definitive implementation of telemedicine in health systems, requires multifaceted approaches. The development of digital platforms followed by rigorous research, such as that carried out in the present study, will be crucial to filling existing gaps in scientific knowledge, especially about cost-effectiveness, leadership roles, and the impact of telemedicine in different regions of the planet. Doctors must adapt to this new paradigm, while political decision-makers play a critical role in improving infrastructure, promoting digital literacy, and establishing policies based on scientific evidence [23]. The joint effort between health systems and public policies can promote a comprehensive transformation, establishing a global reference aimed at the definitive implementation of digital innovation in the provision of services and health care for the population [27].

The COVID-19 pandemic has provided valuable insights for the future of virtual care. To ensure its long-term sustainability, it is crucial to design robust platforms, implement supportive policies, and continuously evaluate its effectiveness. These efforts should focus on addressing technological challenges, minimizing digital exclusion, and optimizing patient experiences [28].

Conversely, we observed possible limitations to the study. One of them is the cultural capacity of the population studied to handle digital equipment. Certainly, not everyone had the skills to do so. Although this technology has shown benefits during the pandemic, challenges such as technological limitations and the need for qualified professionals persist for the widespread implementation of telemedicine systems. A final potential limitation is the population sample, since telemedicine is not a one-size-fits-all solution.

Other initiatives in Brazil show that a system employing multiple telemedicine approaches led to fewer emergency department visits and hospitalizations, indicating a beneficial effect on overall healthcare use [29]. A telehealth service applied on a large scale in a limited-resource region to tackle COVID-19 can be related to reduced hospitalizations without increasing the mortality rate [30].

## Conclusions

Despite declining COVID-19 cases worldwide due to effective vaccination efforts, physicians must remain prepared for rapid transitions to telemedicine. Successful implementation requires adequate training, education, and access to digital resources for remote care. To our knowledge, this is the first study to examine the use of virtual care in a large, defined population during the COVID-19 pandemic. The E-care telemedicine system effectively reduced in-person visits, saving users time and money while also helping prevent the spread of infectious diseases. Although telemedicine shows promise for managing COVID-19 within university populations, broader implementation depends on addressing significant gaps in both equitable access and infrastructure. As a relatively new component of primary care, telemedicine warrants further research to optimize its use and evaluate its impact on patient care quality and clinical outcomes.

## Supplementary material

The following online material is available for this article:

Additional file 1. Symptom survey based on a COVID-19 decision tree to streamline assessment by nurses and doctors.

## Availability of data and materials

 The data supporting the findings of this study are not publicly available due to privacy or ethical restrictions, although they can be obtained from the corresponding author upon request. The preprint is available at https://ssrn.com/abstract=4938151 or http://dx.doi.org/10.2139/ssrn.4938151
