# Supplementary material for: Telemedicine for COVID-19 management in Brazil: outcomes and health system implications from a prospective cohort study
Source: J Venom Anim Toxins Incl Trop Dis. 2025 Dec 15;31:e20250030. doi: 10.1590/1678-9199-JVATITD-2025-0030 (PMC12707318; doi:10.1590/1678-9199-JVATITD-2025-0030)
Supplement: Additional file 1. [file 1678-9199-jvatitd-31-e20250030-s1.pdf]

J Venom Anim Toxins incl Trop Dis
https://doi.org/10.1590/1678-9199-JVATITD-2025-0030

Supplementary Material to "Telemedicine for COVID-19 management in Brazil: outcomes and health system implications from a prospective cohort study"

Additional file 1. Symptom survey based on a COVID-19 decision tree to streamline assessment by nurses and doctors.

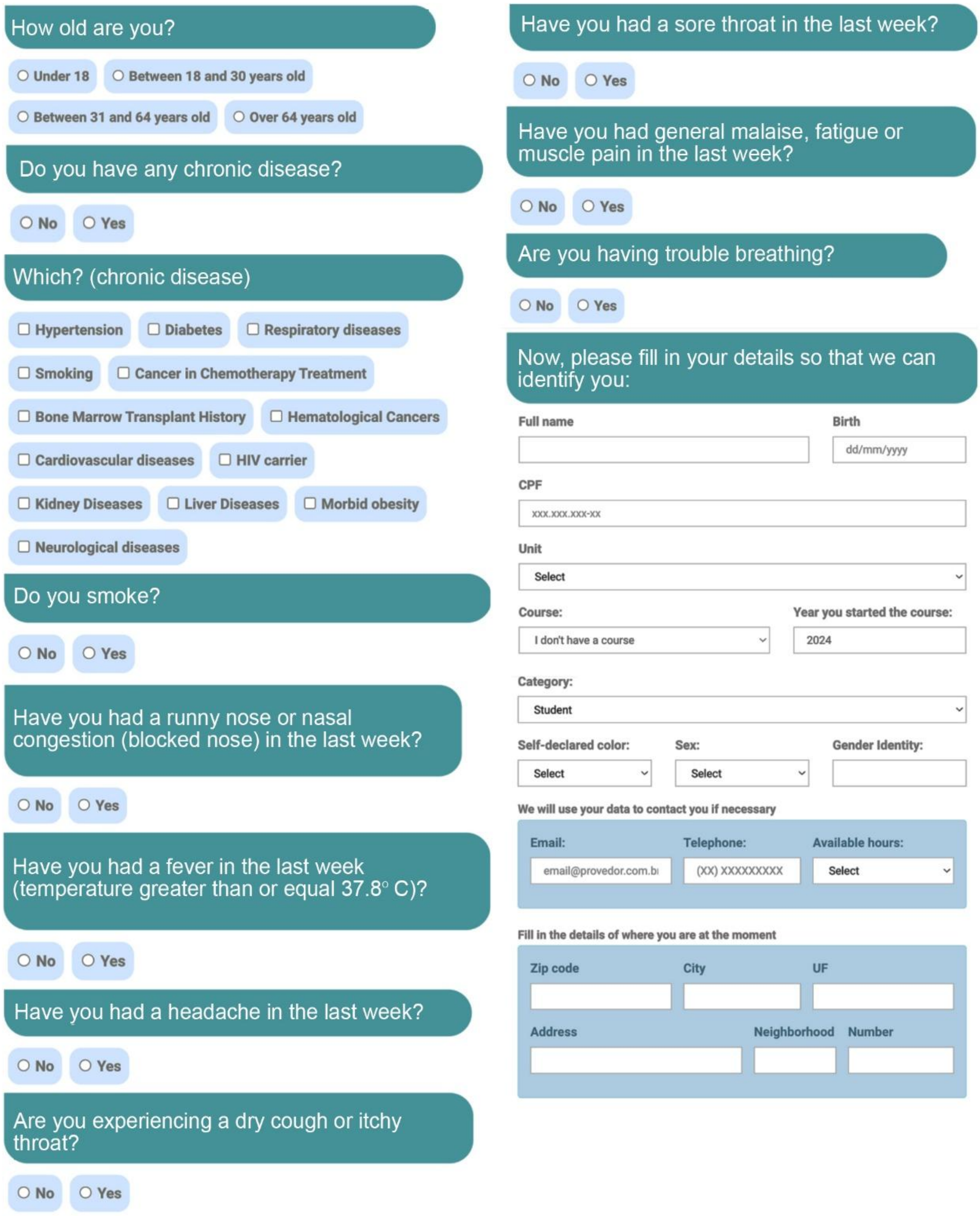
How old are you?
Under 18
Between 18 and 30 years old
Between 31 and 64 years old
Over 64 years old
Do you have any chronic disease?
No
Yes
Which? (chronic disease)
Hypertension
Diabetes
Respiratory diseases
Smoking
Cancer in Chemotherapy Treatment
Bone Marrow Transplant History
Hematological Cancers
Cardiovascular diseases
HIV carrier
Kidney Diseases
Liver Diseases
Morbid obesity
Neurological diseases
Do you smoke?
No
Yes
Have you had a runny nose or nasal congestion (blocked nose) in the last week?
No
Yes
Have you had a fever in the last week (temperature greater than or equal 37.8° C)?
No
Yes
Have you had a headache in the last week?
No
Yes
Are you experiencing a dry cough or itchy throat?
No
Yes
Have you had a sore throat in the last week?
No
Yes
Have you had general malaise, fatigue or muscle pain in the last week?
No
Yes
Are you having trouble breathing?
No
Yes
Now, please fill in your details so that we can identify you:
Full name
Birth
dd/mm/yyyy
CPF
xxx.xxx.xxx-xx
Unit
Select
Course:
I don't have a course
Year you started the course:
2024
Category:
Student
Self-declared color:
Select
Sex:
Select
Gender Identity:
We will use your data to contact you if necessary
Email:
email@provedor.com.br
Telephone:
(XX) XXXXXXXXX
Available hours:
Select
Fill in the details of where you are at the moment
Zip code
City
UF
Address
Neighborhood
Number
